# Crocin Improves Insulin Sensitivity and Ameliorates Adiposity by Regulating AMPK-CDK5-PPAR*γ* Signaling

**DOI:** 10.1155/2020/9136282

**Published:** 2020-06-06

**Authors:** Kai Fang, Ming Gu

**Affiliations:** Department of Pharmacy, Union Hospital, Tongji Medical College, Huazhong University of Science and Technology, Wuhan 430022, China

## Abstract

Crocin is a carotenoid compound which possesses multiple biological activities. Our and other laboratory's previous findings show that crocin alleviates obesity and type 2 diabetes-related complications. We have found that crocin activates AMP-activated protein kinase (AMPK) signaling and inhibition of AMPK suppresses crocin-induced protective effects. However, the causal role of AMPK activation in the biological role of crocin is still not verified. In the present study, we showed that crocin markedly inhibits the changes of glucose metabolic parameters and serum lipid profiles in wild type diabetic mice. In AMPK*α* KO diabetic mice, those protective effects of crocin against glucose and lipid metabolic dysfunction were abolished. These results demonstrated AMPK activation was responsible for the beneficial effects of crocin on metabolic dysfunction. Moreover, we have shown that the antiobese effect of crocin has been abolished by the deficiency of AMPK*α*. We also showed that crocin induced a significant decrease of CDK5 protein level in wild type diabetic mice, while this effect was abolished in AMPK*α* KO diabetic mice. The regulation of downstream targets of CDK5/PPAR*γ* by crocin was abolished by the deficiency of AMPK. In conclusion, our study verified that activation of AMPK is involved in crocin-induced protective effects against glucose and lipid metabolic dysfunction. Activation of AMPK downregulates the protein level of CDK5, followed by the decrease of PPAR*γ* phosphorylation, leading to the inhibition of adipose formation and metabolic dysfunction. Our study provides new insights into the mechanism of protective effects of crocin and interaction of AMPK and CDK5/PPAR*γ* signaling.

## 1. Introduction

The prevalence of obesity and type 2 diabetes (T2D) have become major health problems worldwide [1, 2]. With the increasing number of obese individuals associated with the aging of population, the prevalence of T2D and other associated complications is increasing at an unexpected rate. However, there is still no curative pharmacological treatment for obesity and type 2 diabetes.

Crocin is a water-soluble carotenoid compound and a main active constituent found in the stigmas of Crocus sativus, commonly known as saffron [3, 4]. Crocin was used flavoring and coloring agent in food manufacture [5]. Crocin has been found to possess multiple pharmacological effects, including antioxidant, antihyperlipidemic, anti-inflammatory, anticancer, antiarthritic, hepatoprotective, and cardioprotective effects [6–8]. Our and other laboratory's findings show that crocin alleviates obesity and type 2 diabetes-related complications [9–13]. We have found that crocin activates AMP-activated protein kinase (AMPK) signaling and inhibition of AMPK significantly suppresses crocin-induced protective effects against metabolic disorders [9, 10]. However, the causal role of AMPK activation in the biological role of crocin is still not verified, and the AMPK-associated downstream signaling pathway mediating the beneficial effect of crocin is still not known.

Peroxisome proliferator-activated receptor gamma (PPAR*γ*) is a pivotal nuclear receptor and transcription factor that plays substantial roles in the regulation of glucose and lipid metabolism through the regulation of gene expression [14, 15]. The chemical ligands have been developed into therapeutic drugs for the treatment of type 2 diabetes. In addition to the ligand activation, PPAR*γ* can also be regulated through diverse posttranslational modifications [16]. In recent years, it has been found that PPAR*γ* serine 245 (or S273 in PPAR*γ* isoform 2) can be phosphorylated by cyclin-dependent kinase 5 (CDK5), and this posttranslational modification is related with insulin resistance in obese individuals [17]. However, no evidence has shown whether crocin could regulate CDK5/PPAR*γ* signaling.

In the present study, we designed experiments to evaluate the causal role of AMPK in the protective effects of crocin against metabolic disorders and to investigate the possible effects of crocin on CDK5/PPAR*γ* signaling.

## 2. Materials and Methods

### 2.1. Animal Experiments

All animal experiments were approved by the Institutional Animal Care and Use Committee of Union Hospital, Tongji Medical College, Huazhong University of Science and Technology and were conducted in accordance with ARRIVE and NIH guidelines for animal welfare. Male mice with global knockout of AMPK*α*2 gene (AMPK*α*2-KO) were purchased from The Jackson Laboratory (Bar Harbor, Maine, USA). Wild-type (WT) mice with C57BL/6 J background were used as control.

All mice were housed in the University of Louisville Research Resources Center with a constant temperature at 22°C and a 12-h light/dark cycle. Mice had free access to tap water. The model of T2D was established by high-fat diet (HFD; 60.3 kcal% fat, TD. 09766; Research Diets, Teklad Custom, Envigo) feeding for three months to induce insulin resistance, followed by a single intraperitoneal injection of 100 mg/kg streptozotocin (STZ; Sigma-Aldrich) in 0.1 mol/L of citrate acid buffer (pH 4.5) to induce partially impaired *β*-cell function. The dose of STZ was used according to previous reports [18]. The survival rates after STZ injection in wild type and AMPK*α*2-KO mice were 93.3% and 86.7%, respectively. 7 days post-STZ injection, mice with hyperglycemia (3-h fasting blood glucose levels ≥250 mg/dL) were defined as diabetic. The diabetic mice were daily orally administrated with 20 mg/kg crocin for 12 weeks. After the experiment, all mice were fasted for 12 h and anesthetized using 2% isoflurane and sacrificed. Blood samples were collected, and tissue samples were snap-frozen in liquid nitrogen or collected for paraffin embedding.

### 2.2. Histopathology of Adipose Tissue

Histopathology of adipose tissue was observed using H&E staining. In brief, adipose tissue was fixed in 10 formaldehyde, embedded in paraffin, and then 5 *μ*m sections were cut and stained with H&E. Capture of images was performed using a light microscope (Olympus, Japan).

### 2.3. Biochemical Detection

Blood was centrifuged at 2500 rpm for 10 min at 4°C, and serum was collected. Serum levels of triglycerides (TG), nonesterified fatty acids (NEFA), total cholesterol (TC), aspartate aminotransferase (AST), and alanine transaminase (ALT) were quantified using commercial kits (Nanjing Jiancheng, Nanjing, China). Insulin levels in serum were measured using an enzyme-linked immunosorbent assay (ELISA) kit (Invitrogen, Carlsbad, CA, USA) according to the manufacturer's instructions.

### 2.4. Glucose and Insulin Tolerance

11 weeks after the treatment of crocin, intraperitoneal glucose tolerance test (IPGTT) and intraperitoneal insulin tolerance test (IPITT) were performed to determine the ability of the body to respond to glucose or insulin load. Before the IPGTT and IPITT, mice were fasted for 12 h or 6 h, respectively. Blood glucose level was measured 0, 30, 60, 90, and 120 min after the administration of glucose and insulin using an Accu-Chek glucometer (Roche, Basel, Switzerland) via tail vein blood.

### 2.5. Real-Time Quantitative PCR

Total RNA from adipose tissues was extracted using TRIzol reagent (Life Technologies, Carlsbad, CA, USA) as per the manufacturer's instructions. RNA quantity and quality were confirmed using Nanodrop (Thermo Fischer Scientific, USA). After treating with DNase, reverse transcription into cDNA was performed using Superscript II (Life Technologies, Foster City, CA). Real-time RT-PCR reaction was prepared using SYBR Green PCR Master Mix (Takara, Tokyo, Japan) in an ABI StepOnePlus Real-time PCR System. Expression of glyceraldehyde 3-phosphate dehydrogenase (GAPDH) was used to normalize the expression of target genes used in the study.

### 2.6. Western Blot Analysis

Adipose tissues were lysed in RIPA buffer (Beyotime, Jiangsu, China) containing protease inhibitor cocktail. The lysates were centrifuged at 12000 rpm for 20 min at 4°C, and supernatants were collected and used for protein concentration determination using the BCA Protein Assay Kit (Beyotime, Jiangsu, China). An equal amount of protein samples (50 *μ*g) were separated on 10% SDS-polyacrylamide gel and transferred to polyvinylidene fluoride membranes (PVDF, Millipore). After blocking with 5% nonfat dried milk, membranes were then incubated with primary antibodies overnight at 4°C. The primary antibodies included *β*-actin (dilution 1 : 1000; Cell signaling technology), PPAR*γ* (dilution 1 : 1000; Cell signaling technology), p-PPAR*γ* (dilution 1 : 1000; Rockland), and CDK5 (dilution 1 : 1000; Abcam). After that, primary antibody probed membranes were washed three times with TBST for 10 min each. The membranes were then incubated with horseradish-peroxidase (HRP)-conjugated secondary antibody (diluted 1 : 5,000; Thermo Fisher Scientific, USA) for 1 h at room temperature. After probing with secondary antibodies, membranes were rewashed three times. Finally, the bands were visualized using chemiluminescence (ECL) detection reagents (Thermo Fisher Scientific, USA).

### 2.7. Statistical Analysis

Results are expressed as the mean ± SD. Significant differences among groups were assessed using one- way ANOVA followed by Dunnett's test. Statistical significance was defined as *P* < 0.05.

## 3. Results

### 3.1. Deficiency of AMPK*α* Abolished Crocin-Induced Protective Effects on General Biochemical Profiles in Type 2 Diabetic Mice

**Figure 1:**
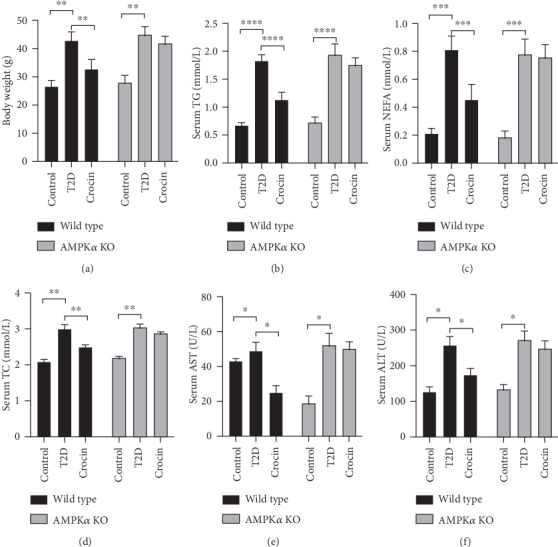
Deficiency of AMPK*α* abolished crocin-induced protective effects on general biochemical profiles in type 2 diabetic mice. Type 2 diabetes was induced in wild type and AMPK*α* mice using high-fat diet incorporated with STZ injection. The type 2 diabetic mice were treated with crocin or vehicle. (a) Body weight (*n* = 6). (b) Serum TG level (*n* = 6). (c) Serum NEFA level (*n* = 6). (d) Serum TC level (*n* = 6). (e) Serum AST activity (*n* = 6). (f) Serum ALT activity (*n* = 6). ∗*P* < 0.05, ∗∗*P* < 0.01, ∗∗∗*P* < 0.005, ∗∗∗∗*P* < 0.001.

To evaluate the causal role of AMPK activation in the protective effects of crocin in the context of obesity and type 2 diabetes, we established obese and type 2 diabetic animal models using AMPK*α* global KO mice. As illustrated in Figure 1(a), the treatment of crocin significantly reduced the body weight in wild type diabetic mice, while this effect was abolished in AMPK*α* KO diabetic mice. We then determined the effect of crocin on general biochemical profiles in both wild type and AMPK*α* KO diabetic mice. We showed that in wild type diabetic mice, crocin significantly reduced the serum levels of triglycerides (TG), nonesterified fatty acids (NEFAs), total cholesterol (TC), and aspartate aminotransferase (AST) and alanine transaminase (ALT) (Figures 1(b)–1(f)). In contrast, in AMPK*α* KO diabetic mice, crocin did not show any inhibitory effects on serum levels of TG, NEFAs, TC, AST, and ALT (Figures 1(b)–1(f)).

### 3.2. Deficiency of AMPK*α* Abolished Crocin-Induced Protective Effects on Glucose Metabolic Activity in Type 2 Diabetic Mice

**Figure 2:**
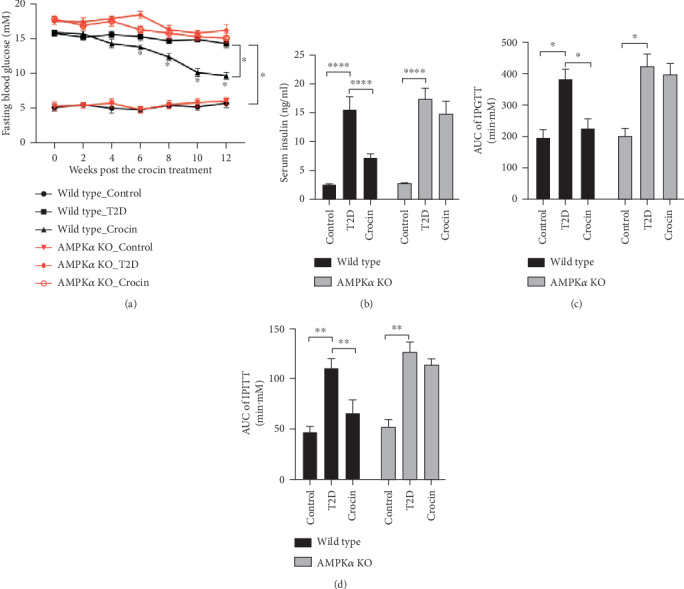
Deficiency of AMPK*α* abolished crocin-induced protective effects on glucose metabolic activity in type 2 diabetic mice. Type 2 diabetes was induced in wild type and AMPK*α* mice using high-fat diet incorporated with STZ injection. The type 2 diabetic mice were treated with crocin or vehicle. (a) Fasting blood glucose level (*n* = 6). (b) Serum insulin level (*n* = 6). (c) Area curve under the curve of IPGTT (*n* = 6). (d) Area curve under the curve of IPITT (*n* = 6). ∗*P* < 0.05, ∗∗*P* < 0.01, ∗∗∗∗*P* < 0.001.

We further determined the effect of crocin on glucose metabolism in both wild type and AMPK*α* KO diabetic mice. As shown in Figure 2(a), fasting blood glucose began to reduce after 6-week treatment of crocin, and at the end of the experiment, fasting blood glucose in wild type diabetic mice was markedly inhibited by crocin. However, no marked change of fasting blood glucose in AMPK*α* KO diabetic mice was observed after the treatment of crocin (Figure 2(a)). Moreover, crocin remarkably reduced the level of insulin in wild type diabetic mice, while this effect was abolished in AMPK*α* KO diabetic mice (Figure 2(b)). We also used IPGTT and IPITT to evaluate the ability of the body to respond to glucose and insulin. As shown in Figures 2(c) and 2(d), the inhibitory effect on the increase of area under the curve of IPGTT and IPITT by crocin was abolished when AMPK*α* was deficient.

### 3.3. Deficiency of AMPK*α* Abolished Crocin-Induced Protective Effects on Adiposity in Type 2 Diabetic Mice

**Figure 3:**
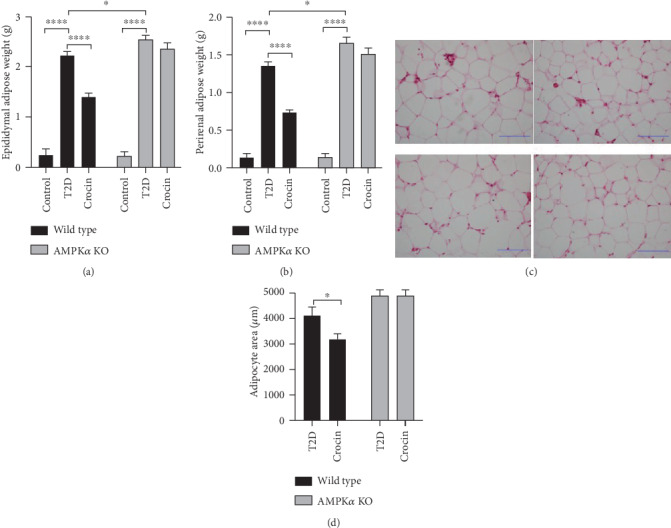
Deficiency of AMPK*α* abolished crocin-induced protective effects on adiposity in type 2 diabetic mice. Type 2 diabetes was induced in wild type and AMPK*α* mice using high-fat diet incorporated with STZ injection. The type 2 diabetic mice were treated with crocin or vehicle. (a) Epididymal adipose weight (*n* = 6). (b) Perirenal adipose weight (*n* = 6). (c) HE staining of adipose tissue (*n* = 6). (d) The size of adipocyte (*n* = 6). ∗*P* < 0.05, ∗∗*P* < 0.01, ∗∗∗∗*P* < 0.001.

Considering the important role of obesity in glucose metabolic dysfunction, we further evaluated the effect of crocin in both wild type and AMPK*α* KO diabetic mice. In Figures 3(a) and 3(b), we showed that in wild type diabetic mice, crocin markedly reduced the perirenal and epidydimal adipose mass, while this effect of crocin on adipose tissue was abolished in AMPK*α* KO diabetic mice. Moreover, crocin reduced the size of adipocyte in wild type diabetic mice, which effect was not observed in AMPK*α* KO diabetic mice (Figures 3(c) and 3(d)).

### 3.4. Deficiency of AMPK*α* Abolished Crocin-Induced Effects on CDK5/PPAR*γ* in Type 2 Diabetic Mice

**Figure 4:**
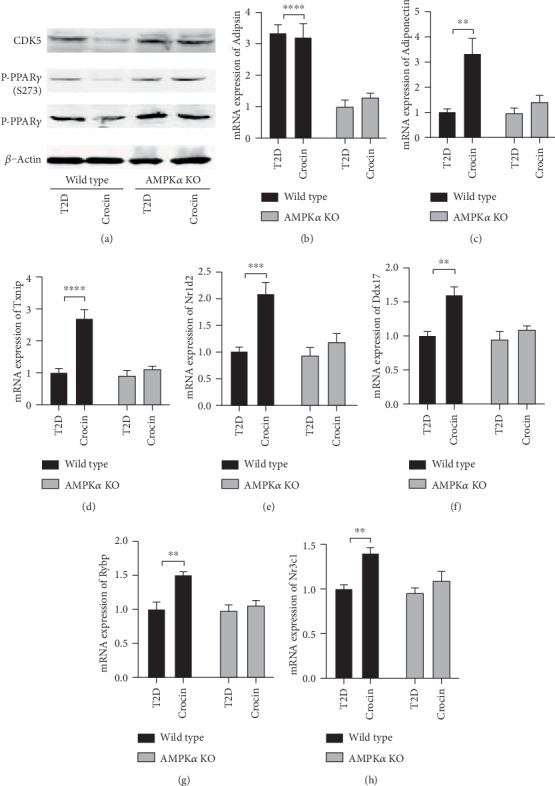
Deficiency of AMPK*α* abolished crocin-induced effects on CDK5/PPAR*γ* in type 2 diabetic mice. Type 2 diabetes was induced in wild type and AMPK*α* mice using high-fat diet incorporated with STZ injection. The type 2 diabetic mice were treated with crocin or vehicle. (a) CDK5 expression and phosphorylation of PPAR*γ* in adipose tissue were determined using western blot (*n* = 6). (b–h) mRNA expression of CDK5/PPAR*γ* targets was determined using RT-qPCR (*n* = 6). ∗*P* < 0.05, ∗∗*P* < 0.01, ∗∗∗*P* < 0.005, ∗∗∗∗*P* < 0.001.

To investigate the possible mechanism of AMPK-mediated protective effects of crocin against obesity and type 2 diabetes, we evaluated the changes of CDK5/PPAR*γ* signaling. Previous literature has demonstrated that CDK5 can phosphorylate PPAR*γ* and thus influence the downstream target genes expression, leading to the regulation of lipid metabolism [19]. We showed that in wild type diabetic mice, crocin significantly reduced the protein level of CDK5 and phosphorylated PPAR*γ* (S273) (Figure 4(a)). However, this effect of crocin on the protein level of CDK5 and phosphorylation of PPAR*γ* (S273) was abolished by the deficiency of AMPK*α* (Figure 4(a)). Furthermore, we showed that in wild type diabetic mice, the mRNA expression of target genes of CDK5/PPAR*γ* signaling, including adipsin, adiponectin, Txnip, Nr1d2, Ddx17, Rybp, and Nr3c1, was significantly increased by crocin (Figures 4(b)–4(h)). However, in AMPK*α* KO diabetic mice, no significant effect of crocin on the expression of those genes was observed (Figures 4(b)–4(h)).

## 4. Discussion

Our previous results have shown that crocin ameliorates glucose and lipid metabolic dysfunction in vivo and in vitro [9, 10]. Using pharmacological inhibitors in mice and genetic manipulation in cells, we have found that AMPK activation plays a substantial role in the beneficial effect of crocin [9, 10]. However, the causal role of AMPK in the biological and pharmacological activities of crocin is still not clear, and the downstream signaling of AMPK is still not completely understood.

In the present study, our goal is to define the causal role of AMPK activation in the protective effects of crocin against metabolic disorders using AMPK*α* KO mice. We used high-fat diet and STZ to induce obese and type 2 diabetic models. Our results showed that crocin markedly inhibits the changes of glucose metabolic parameters and serum lipid profiles in wild type diabetic mice. In AMPK*α* KO mice, those protective effects of crocin against glucose and lipid metabolic dysfunction were abolished. These results demonstrated AMPK activation was responsible for the beneficial effects of crocin on metabolic dysfunction. Moreover, we have shown that the antiobese effect of crocin has been abolished by the deficiency of AMPK*α*. Activation of AMPK is at the center of the proposed mechanism of metformin's action, which is the first-line drug for the treatment of type 2 diabetes [20]. AMPK is a heterotrimeric protein with *α*, *β*, and *γ* subunits. AMPK activation has been verified to enhance cellular glucose uptake and inhibit intracellular glucose production [21]. AMPK activity is reduced under both obese and type 2 diabetic conditions [22, 23]. It has been suggested that AMPK plays an important role in the regulation of adipose dynamics through increasing basal lipolysis restraining *β*-adrenergic-induced lipolysis [24]. All these results suggest that AMPK is a pivotal target of crocin which mediates the protective effect on glucose and lipid metabolic dysfunction.

PPAR*γ* is an important transcription factor that plays substantial roles in the regulation of glucose and lipid metabolism which is also a target of pharmacological therapy of diabetes [14, 15]. PPAR*γ* is critical for the adipogenesis through regulating a battery of genes responsible for triglyceride synthesis [25]. However, the interaction between PPAR*γ* and AMPK is still not clear. In this study, we found that crocin significantly inhibited the phosphorylation of PPAR*γ* in wild type diabetic mice, while this effect was abolished in AMPK*α* KO diabetic mice. The data suggest that AMPK activation is crucial for crocin-induced inhibition of the phosphorylation of PPAR*γ*. This finding provides a link between the regulation of AMPK and PPAR*γ*.

In the literature, CDK5 is reported to regulate posttranslational modifications of PPAR*γ* [16]. Phosphorylation of PPAR*γ* at serine 273 by CDK5 has been shown to stimulate diabetogenic gene expression in adipose tissues [17]. The antidiabetic PPAR*γ* ligand drugs, such as the thiazolidinediones and PPAR*γ* partial/nonagonists, can inhibit this modification, leading to the therapeutic improvement [26]. In our study, we showed that crocin resulted in a significant decrease of CDK5 protein level in wild type diabetic mice, while this effect was abolished in AMPK*α* KO diabetic mice. The regulation of downstream targets of CDK5/PPAR*γ* by crocin was abolished by the deficiency of AMPK. The results suggest that the downregulation of CDK5 might be involved in the regulation of PPAR*γ* induced by crocin, and this effect is mediated by the activation of AMPK.

In this study, we confirmed the beneficial effects of crocin on liver injury, adiposity, obesity, and insulin resistance. These results suggest that crocin could have the potential to prevent diabetes-associated liver injury. Crocin is also a promising agent in preventing obesity and insulin resistance. Based on the crucial roles of phosphorylation of PPAR*γ* and PPAR*γ* signaling in the regulation of insulin signaling and adipocyte differentiation, we propose that AMPK-mediated inhibition of CDK5 and thus repression of PPAR*γ* phosphorylation and activation is a pivotal step for crocin-induced beneficial effects. We have used AMPK*α* KO mice to confirm the causal role of AMPK activation in the protective effects of crocin. However, further studies are still needed to verify whether CDK5/PPAR*γ* signaling is the unique downstream pathway and essential for crocin-induced protective effects.

In conclusion, our study verified that activation of AMPK is involved in crocin-induced protective effects against glucose and lipid metabolic dysfunction. Activation of AMPK downregulates the protein level of CDK5, followed by the decrease of PPAR*γ* phosphorylation, leading to the inhibition of adipose formation and metabolic dysfunction. Our study provides new insights into the mechanism of protective effects of crocin and the interaction of AMPK and CDK5/PPAR*γ* signaling.

## Data Availability

The data will be available on request.
